# Proposing and investigating PCAMARS as a novel model for NO_2_ interpolation

**DOI:** 10.1007/s10661-019-7253-2

**Published:** 2019-02-23

**Authors:** Mohsen Yousefzadeh, Mahdi Farnaghi, Petter Pilesjö, Ali Mansourian

**Affiliations:** 1 Faculty of Geodesy and Geomatics Engineering, K. N. Toosi University of Technology, Tehran, Iran; 2 GIS Center, Department of Physical Geography and Ecosystem Science, Lund University, 22362 Lund, Sweden; 3 Center for Middle-Eastern Studies, Lund University, Lund, Sweden

**Keywords:** Air pollution, Spatial interpolation, MARS, PCA, NO_2_

## Abstract

Effective measurement of exposure to air pollution, not least NO_2_, for epidemiological studies along with the need to better management and control of air pollution in urban areas ask for precise interpolation and determination of the concentration of pollutants in nonmonitored spots. A variety of approaches have been developed and used. This paper aims to propose, develop, and test a spatial predictive model based on multivariate adaptive regression splines (MARS) and principle component analysis (PCA) to determine the concentration of NO_2_ in Tehran, as a case study. To increase the accuracy of the model, spatial data (population, road network and point of interests such as petroleum stations and green spaces) and meteorological data (including temperature, pressure, wind speed and relative humidity) have also been used as independent variables, alongside air quality measurement data gathered by the monitoring stations. The outputs of the proposed model are evaluated against reference interpolation techniques including inverse distance weighting, thin plate splines, kriging, cokriging, and MARS3. Interpolation for 12 months showed better accuracies of the proposed model in comparison with the reference methods.

## Introduction

NO_2_ has adverse effect on human life and environment. Numerous studies have shown the effect of NO_2_ exposure on respiratory problems and deterioration of asthmatic patients (Pollution 2010). NO_2_ is also one of the main causes of acidification of soil and eutrophication of lakes (Hedley and Bolan 2003; Bouwman et al. 2002). In order to protect vulnerable people and reduce environmental damages, reliable data/maps about the NO_2_ concentration, especially in urban areas, are needed (Briggs et al. 1997).

Creating dense network of air quality stations to measure NO_2_ concentration is not cost effective. So, either of the following two approaches are used to calculate and/or estimate NO_2_ concentrations. The first approach is based on the classical dispersion models. These models use the laws of physics and determine the NO_2_ concentration in a vicinity as a function of meteorology, street geometry, receptor locations, traffic volumes, and emission factors (Zheng et al. 2013; Vardoulakis et al. 2003). Dispersion models are usually based on empirical assumptions and parameters that might not be applicable to all urban environments. For example, they may require the roughness coefficient of the urban surfaces and the gaps between buildings, which are challenging to be obtained precisely for a large area. Therefore, such models are not efficient to be used in large scale (Zheng et al. 2013).

The second approach is to use interpolation methods to determine NO_2_ concentrations in an area based on the values measured by air pollution monitoring stations. Various techniques have been used for air pollution interpolation, including deterministic methods (e.g., IDW (inverse distance weighting) (Bell 2006), RBF (radial basis function) (Deligiorgi and Philippopoulos 2011), nearest-neighbor and polynomial methods (Isaaks and Srivastava 1989b)) and stochastic methods (e.g., simple kriging (Wong et al. 2004), ordinary kriging (OK) (Janssen et al. 2008), kriging with external drift (Pearce et al. 2009), and universal kriging (Jerrett et al. 2005)). The problem with these conventional techniques is that their performance is heavily affected by the number and spatial distribution of available monitoring stations (Singh et al. 2011). In addition, previous studies show that air pollution concentration in urban areas varies by location, nonlinearly, and depends on multiple factors such as meteorology, traffic, land use, and urban structure (Zheng et al. 2013; Vardoulakis et al. 2003). Also, air pollution at any point is affected by the density of air pollution in the surrounding areas (Dong and Liang 2014; Hao and Liu 2016). These issues are rarely addressed in the conventional interpolation models.

In order to address the shortcomings of the conventional methods, various interpolation techniques have been proposed in the literature. Among them, cokriging (CK) and multivariate adaptive regression splines (MARS) have been successfully applied on air pollution interpolation problem. In CK approach, additional data are provided and added to the interpolation calculations as secondary variables (Singh et al. 2011; Isaaks and Srivastava 1989a). Additionally, it exploits both the autocorrelations and cross-correlations among all involved variables including the target variable and the predictor variables. Despite its benefits, it is not practical to use more than two or three secondary variables in CK, due to computational complexity (Wang et al. 2013). MARS is another approach that has been used to improve the accuracy of interpolation. In a study by Shahraiyni et al. (2015), air pollutants have been interpolated using MARS and the performance is compared with IDW, TPSS (thin plate splines), kriging, and CK. Their MARS model utilizes latitude, longitude, and elevation, as independent variables.

The main goal of this study is to increase the accuracy of interpolation of NO_2_ pollutant based on the measurements of air pollution monitoring stations by adding several predictor variables to MARS. However, the main challenge is that when a large number of predictor variables are introduced to MARS, the model cannot adjust well and overfits (Kartal Koc and Bozdogan 2015). This situation even worsens when MARS is going to be used for solving an interpolation problem, like air pollution interpolation, with limited number of sample points in a large study area.

In order to increase the accuracy of interpolation and generating high-resolution maps of NO_2_, this study develops and suggests a new model called PCAMARS which is an extension to MARS by PCA (principal component analysis). PCAMARS provides the possibility of using multiple secondary parameters for the interpolation of air pollution concentration. The proposed method in this study, in addition to the monitored NO_2_ data, gathered by air pollution monitoring stations, uses meteorological, topographical, and urban data as auxiliary inputs. It also takes the spatial effect into account by considering the spatial correlation between NO_2_ and the secondary variables.

PCAMARS was implemented and tested in Tehran (the capital of Iran), which has substantial air pollution problems, as case study area. The results of PCAMARS have been compared with IDW, TPSS, OK, CK, and MARS.

## Theory

The presented interpolation method in this study has been developed based on MARS and PCA. The basics of the two methods are briefly described in this section.

### MARS

MARS, as a nonlinear and nonparametric regression method, was first introduced by Friedman (Friedman 1991) in 1991. MARS models nonlinear interaction between the inputs and the output of a system using a series of piecewise linear segments (splines) of different gradients (Zhang and Goh 2013). These splines are known as basis functions (BFs), which can be considered either linear or cubic (for simplicity, only the piecewise linear function is described here). The end points of the segments are called knots. A knot marks the end of one region of data and the beginning of another (Zhang and Goh 2013). The result of using such a structure brings high flexibility to MARS that can handle both linear and nonlinear behavior (Zhang and Goh 2016).

MARS aims to model a function, of *y* = *f*(*x*), where *x* = (*x*_1_, *x*_2_, *x*_3_, ⋯, *x*_*m*_, ⋯, *x*_*p*_) is the vector of *p* input variables and *y* is the output variable in the form of Eq. (1), as the weighted sum of piecewise linear BF, *B*_*i*_, where each *c*_*i*_ is a constant coefficient and *c*_0_ is the intercept.

1 $$ \widehat{f}(X)=\sum \limits_{i=1}^k{c}_i{B}_i(X)+{c}_0 $$

MARS generates BFs by stepwise searching through an adaptive regression algorithm (Zhang and Goh 2016). The MARS implementation procedure consists of two phases, including a forward phase and a backward phase. The forward phase creates an initial collection of BFs in the form of Eq. (1). In this phase, the range of output variable is partitioned into several groups, where for each partition, a separate BF is considered in the form of *c*_*i*_*B*_*i*_(*X*). The forward phase tries to find the best possible location for the knots by minimizing the sum of squares error (SSE) of the overall model (Rounaghi et al. 2015). The first phase normally results in an over-fit model. Then, the backward phase prunes the least effective BFs (Zhang and Goh 2013).

The backward step starts with the over-fit model, $\widehat{f}(X)$ with *m* BFs, resulted from the first step as input and iteratively eliminates a BF from the current model to create models with *m* − 1, *m* − 2, … , 2, 1, 0 BFs, respectively. In each iteration, a BF whose removal will result in the minimum increase in the overall SSE is eliminated. Eventually, the model with the lowest Generalized Cross Validation (GCV) value will be selected as the final MARS model (Shahraiyni et al. 2015). The GCV equation is a goodness-of-fit test that penalizes large number of BFs and serves to reduce the chance of overfitting. GCV is defined as Eq. (2), where *m* is the number of BFs, *d* is penalizing parameter (the penalty for each basis function), *n* is the number of observation, and *f*(*x*_*i*_) denotes the predicted values of the MARS model (Zhang and Goh 2013). It can be said that *d* is a smoother variable that controls the trade-off between simple and complex models (Rounaghi et al. 2015).

2 $$ \mathrm{GCV}=\frac{\raisebox{1ex}{$1$}\!\left/ \!\raisebox{-1ex}{$n$}\right.{\sum}_{i=1}^n{\left[{y}_i-f\left({x}_i\right)\right]}^2}{{\left[1-\frac{m+d\times \left(m-1\right)/2}{n}\right]}^2} $$

### Principle component analysis

High dimensional input space, correlation among variables, and scarcity of training samples can cause problems for the learning processes (Juhos et al. 2008). This problem, particularly when the goal is to spatially interpolate values for many locations within a city based on few observation points, can be exacerbated and even in some cases, it can prevent the model from proper training. Dimension reduction methods can be used to reduce many correlated variables into a number of uncorrelated variables.

The dimension reduction by PCA leads to transformation of the input variables into a set of new uncorrelated variables known as the principal components, while trying to maintain the maximum variation and dispersion in the data. Equations (3) and (4) define the linear transformation from the input space to the principal component space, where *P* is an orthogonal linear transformation matrix, *Z* is the matrix of original data in which each row represents a variable, and *Y* is a matrix of transformed variables where each row represents an uncorrelated principle components (Markhvida et al. 2018).

3 $$ PZ=Y $$

4 $$ \left[\begin{array}{ccc}{p}_{1,{T}_1}& \cdots & {p}_{1,{T}_m}\\ {}\vdots & \ddots & \vdots \\ {}{p}_{m,{T}_1}& \cdots & {p}_{m,{T}_m}\end{array}\right]\left[\begin{array}{ccc}{z}_{T_1}\left({x}_1\right)& \cdots & {z}_{T_1}\left({x}_n\right)\\ {}\vdots & \ddots & \vdots \\ {}{z}_{T_m}\left({x}_1\right)& \cdots & {z}_{T_m}\left({x}_n\right)\end{array}\right]=\left[\begin{array}{ccc}{y}_1\left({x}_1\right)& \cdots & {y}_1\left({x}_n\right)\\ {}\vdots & \ddots & \vdots \\ {}{y}_m\left({x}_1\right)& \cdots & {y}_m\left({x}_n\right)\end{array}\right] $$

The PCA obtains the transformation matrix *P* from the eigenvalues (*λ*_1_, *λ*_2_,  … , *λ*_1_) of the covariance matrix of the original variables. The rows of this matrix *P* are the corresponding eigenvector (Ensor et al. 2017). The eigenvectors (principle components, PCs) determine the directions of the new space, and the eigenvalues determine their magnitude. To decide which eigenvector(s) can be dropped without losing too much information for the construction of the lower-dimensional subspace, we need to inspect the corresponding eigenvalues. The eigenvectors with the lowest corresponding eigenvalues bear the least information about the distribution of the data and can be dropped (Campos et al. 2018).

## Materials and methods

### Case study

**Fig. 1:**
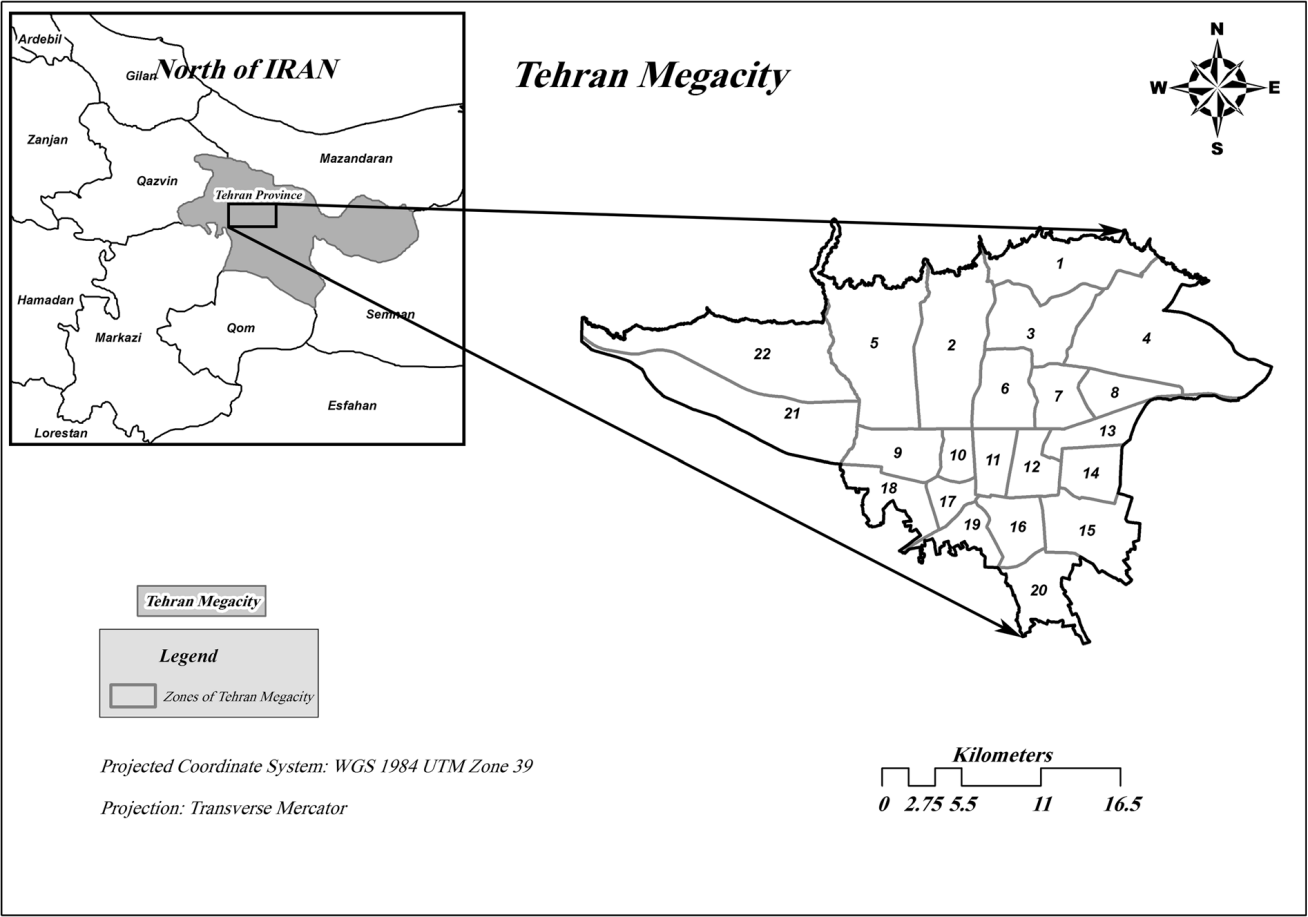
Case study area, Tehran, Iran

The study area, Tehran (the capital of Iran), is located in the northern half of the country (longitude between 35.56 and 35.83 N and latitude between 51.20 and 51.61E) with an area of almost 730 km^2^ (Fig. 1). Tehran has a population of about 8.5 million. In the northern parts, the city reaches to the Alborz Mountains and the rest of the area is covered with hills and in some part with flat plains. The average height of the city in the northern, middle, and southern regions is 1700, 1200, and 1100, respectively.

**Fig. 2:**
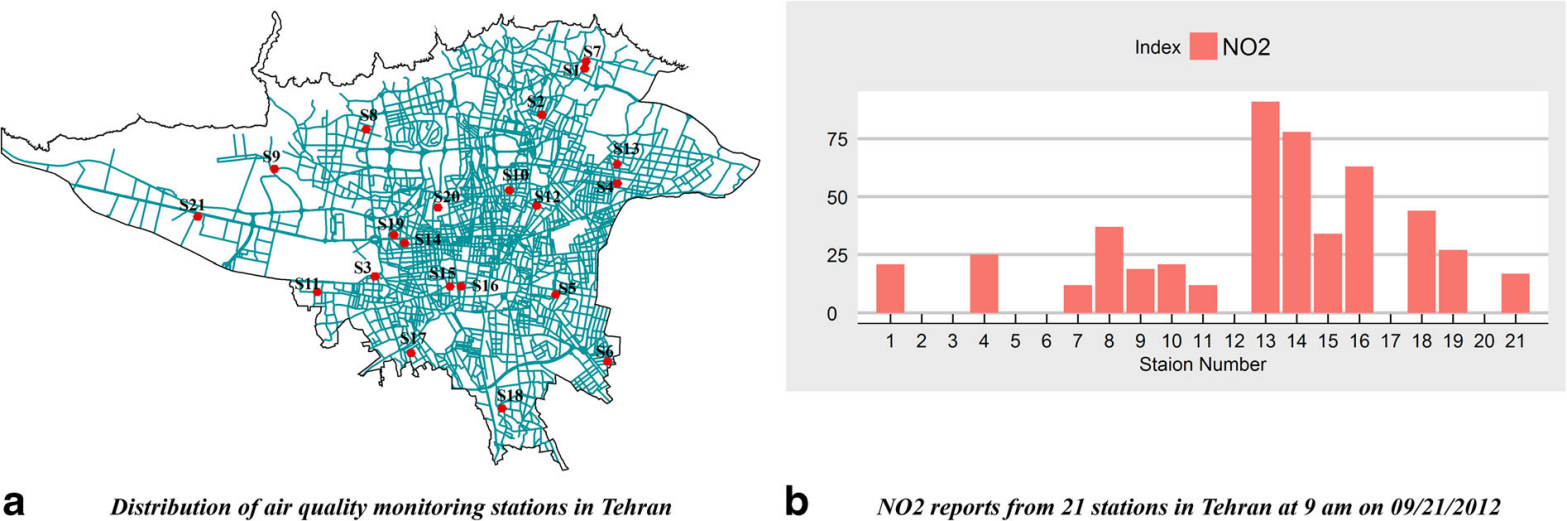
Monitoring stations and spatial variability of NO_2_ in Tehran. **a** Distribution of air quality monitoring stations in Tehran. **b** NO_2_ reports from 21 stations in Tehran at 9 AM on September 21, 2012

Air Quality Control Agency of Tehran municipality has been measuring air pollutants such as CO, NO_2_, SO_2_, O_3_, and PM10 using 21 air pollution monitoring stations (Fig. 2a), and the outputs have been saved as hourly averaged records. In general, spatial heterogeneity in concentrations varies among pollutants and sources (Marshall et al. 2008). As an example, Fig. 2b shows the NO_2_ air pollution concentration in Tehran at 9 AM on September 21, 2012, which illustrates that the emission of NO_2_ in different locations, even among adjacent stations, can vary significantly. The difference between the maximum and the minimum amounts of NO_2_ among stations is more than 50 μg/m^3^. In other words, NO_2_ follows diverse patterns in different locations (e.g., station 1 and station 7) so that even nearby stations may have dissimilar values.

### Data

The hourly averaged observations of NO_2_ of the monitoring at a specific time were used in this study as the dependent variable to be interpolated across the city using the proposed model. The model, in addition to the NO_2_ observations at the specific time, exploits some independent variables to increase the accuracy of the interpolation. Meteorological data, elevation, POI (point of interest), road network structure, and population, which represent the dynamism of urban areas (Zheng et al. 2013; Honarvar and Sami 2018; Yu et al. 2016; Zheng et al. 2015), were used as independent variables.

The meteorological conditions often have direct effect on the local air quality in urban environment through accumulation or ventilation of pollutants and regional transport of clean or polluted air (Seo et al. 2018). Therefore, meteorological observations, including air pressure, temperature, relative humidity, and wind speed, were collected from Iran Meteorological Organization and were used as input variables. Elevation has also considerable influence on the air pollution patterns (Zheng et al. 2013), especially in hilly cities like Tehran. In this regard, digital evaluation model of Tehran was used as another independent variable in this study.

Additionally, road network, as an indicator of traffic-related pollutants, as well as urban blocks with population data and POIs, as indicators of human activity–related air pollutants, were considered as independent variables in the model. Among them, the category of POIs and their density in a region indicate the land use and the function of the region (Hsieh et al. 2015; Yu et al. 2016; Zheng et al. 2013) which can directly affect the local air pollution. Four classes of POIs, including gas and petrol facilities (having strong positive correlations with NO_2_ pollutant) and green areas and sport fields (having strong negative correlations with NO_2_) were retrieved from Open Street Map and used in the model.

In the proposed model, the data is converted into raster of 500-m resolution. The whole analysis is performed in the same resolution, and finally, the output interpolation map is generated.

### Model

**Fig. 3:**
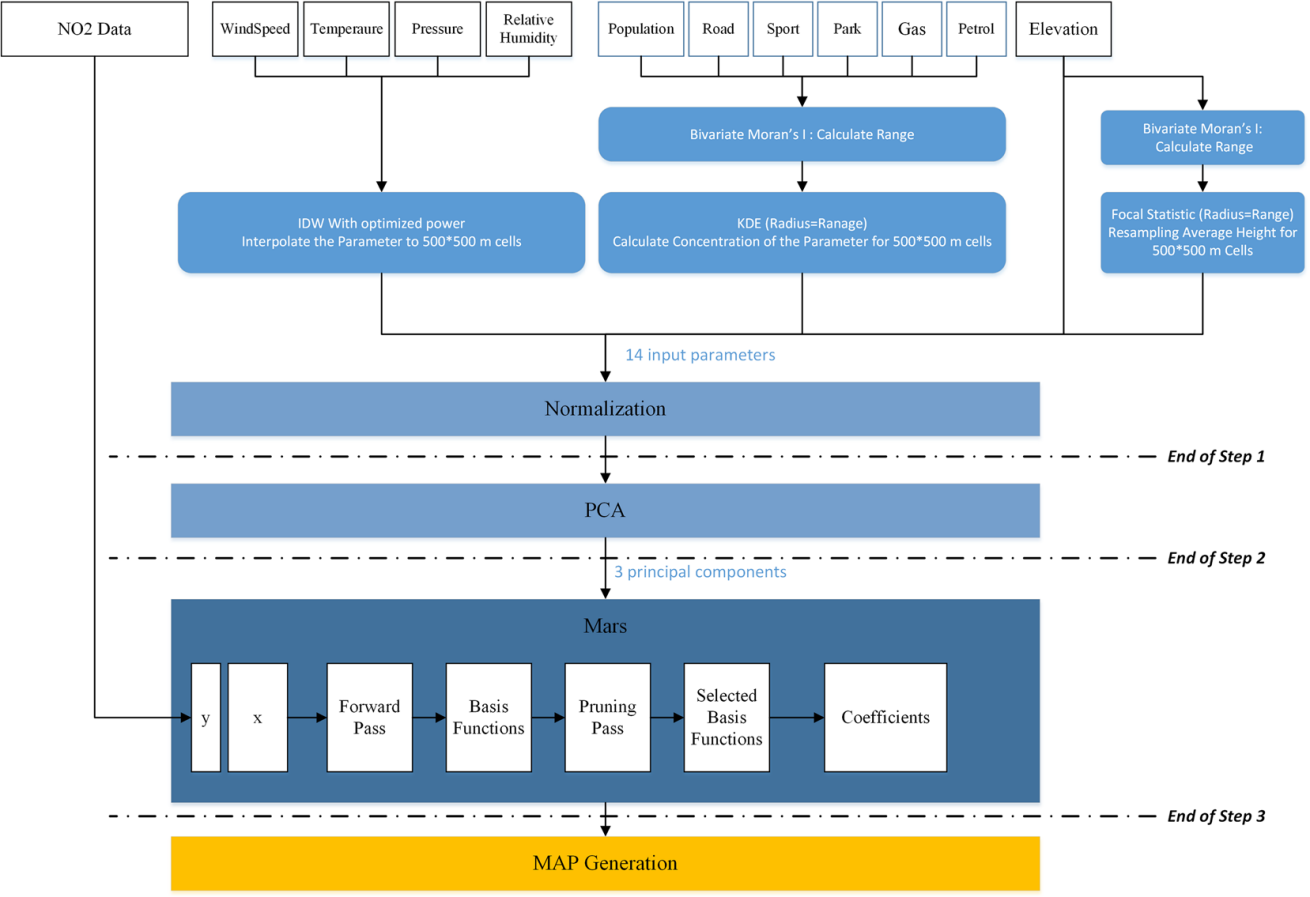
The model architecture

The overall structure of the proposed model for interpolation of NO_2_, called PCAMARS, is shown in Fig. 3. As the figure shows, the average hourly measurement of NO_2_ of the monitoring stations at a specific time together with the respective independent parameters are fed to the model. These data are processed in three steps and eventually the interpolation map of NO_2_ is generated (Fig. 3).

In the first step, the raw data are processed and transformed to the proper structure that is needed for the second step, using GIS analytic methods. This step turns existing vector and raster data into raster layers with the same resolution. Accordingly, the following processes are applied to different data.

- Meteorological parameters including temperature, pressure, relative humidity, and wind speed are collected continuously from meteorological monitoring stations. Therefore, to enter these parameters as independent variables into the model, meteorological data are interpolated into raster data using IDW with optimal power.
- Two independent variables, namely, elevation and average elevation of the region, are extracted from the DEM through resampling and focal statistics, respectively. The proper radius of focal statistics analysis is considered to be equal to the distance that generates the maximum correlation between NO_2_ and the neighborhood elevations at the monitoring stations. To determine the optimal radius, Bivariate Moran’s I index (Hu and Rao 2009) is employed. The model starts with a low radius for neighborhood selection and increases the radius at a regular interval. The radius that maximize the value of bivariate Moran’s I between the dependent and independent variables (in this case, the dependent variable is NO_2_ pollutant and the independent variable is the elevation) will be used as the appropriate radius for focal statistics analysis. The output of focal statistics is the average elevation raster.
- The main roads, POIs, and urban blocks with population are also entered as inputs. The idea is that the density of these parameters at the surroundings of each location significantly influences the air pollution concentration at that location. Therefore, in order to calculate the density of the surrounding roads, POIs, and population at each location, KDE (Kernel Density Estimation) analysis (De Smith et al. 2007; Bailey and Gatrell 1995) was applied on each of these input layers. To calculate KDE, we needed to determine the search radius. The optimal search radius is also calculated by the maximization of the bivariate Moran’s I and a density raster for each input layer is created.

The outputs of the first step are 14 variables, pertaining to 4 meteorological parameters (wind speed, temperature, pressure, and relative humidity), 4 POI density variables (gas station density, petrol station density, parks and green area density, and sport fields density), 2 elevation parameters (average elevation and DEM), population density, and road network density, as well as the coordinates (latitude and longitude) variables. Therefore, at each pixel, 14 input values exist as input to the next step. Since each of the 14 values has different range scales, they are normalized using min–max normalization technique (Hosseini and Kaneko 2011).

In the second step, PCA is used to reduce the dimensionality of the data and extract uncorrelated features from the input variables. The PCA receives the 14 independent variables from the first step and calculates 14 principle components as output. Then, the first three principal components that encompass high percentages of the total variance (in most cases more than 80%) are fed as input to the third step.

In the third step, a MARS model is trained using the value of NO_2_ at the monitoring stations and the three principal components of their respective pixels. The forward pass improves the performance of the model by adding BFs and selecting appropriate place for the knots. This improvement is achieved by lowering the SSE. Then, pruning phase eliminates the least-contributing terms, so that at the end, the final MARS model which has the best GCV is determined. At the end of this step, the interpolation model is ready.

Using the interpolation model, the value of NO_2_ for all the pixels are estimated, based on the 14 independent variables. Having the NO_2_ values for all pixels, the output NO_2_ map of the area is generated.

### Evaluation measure

Leave-One-Out Cross Validation (LOOCV) (Wong et al. 2004) was used in this study to calculate the performance of PCAMARS for interpolation of NO_2_ across the study area. LOOCV removes one of the samples (observations of one of the monitoring stations) from the dataset and trains the model using the remaining samples. While the observation values for NO_2_ at the removed sample point is known (*y*_*i*_), the expected value is calculated from the trained model ($\overline{y_i}$). This process continues for other sample points and finally the root-mean-square error (RMSE) is computed according to Eq. (5).

5 $$ \mathrm{RMSE}={\left[{n}^{-1}{\sum}_{i=1}^n{\left|\left({y}_i-\overline{y_i}\right)\right|}^2\right]}^{1/2} $$

## Results and discussion

In order to demonstrate and evaluate the proposed model, it was implemented and ran in the case study area. For the evaluation purpose, the PCAMARS has been compared with IDW, TPSS, kriging (OK), cokriging (CK), and MARS3.

**Fig. 4:**
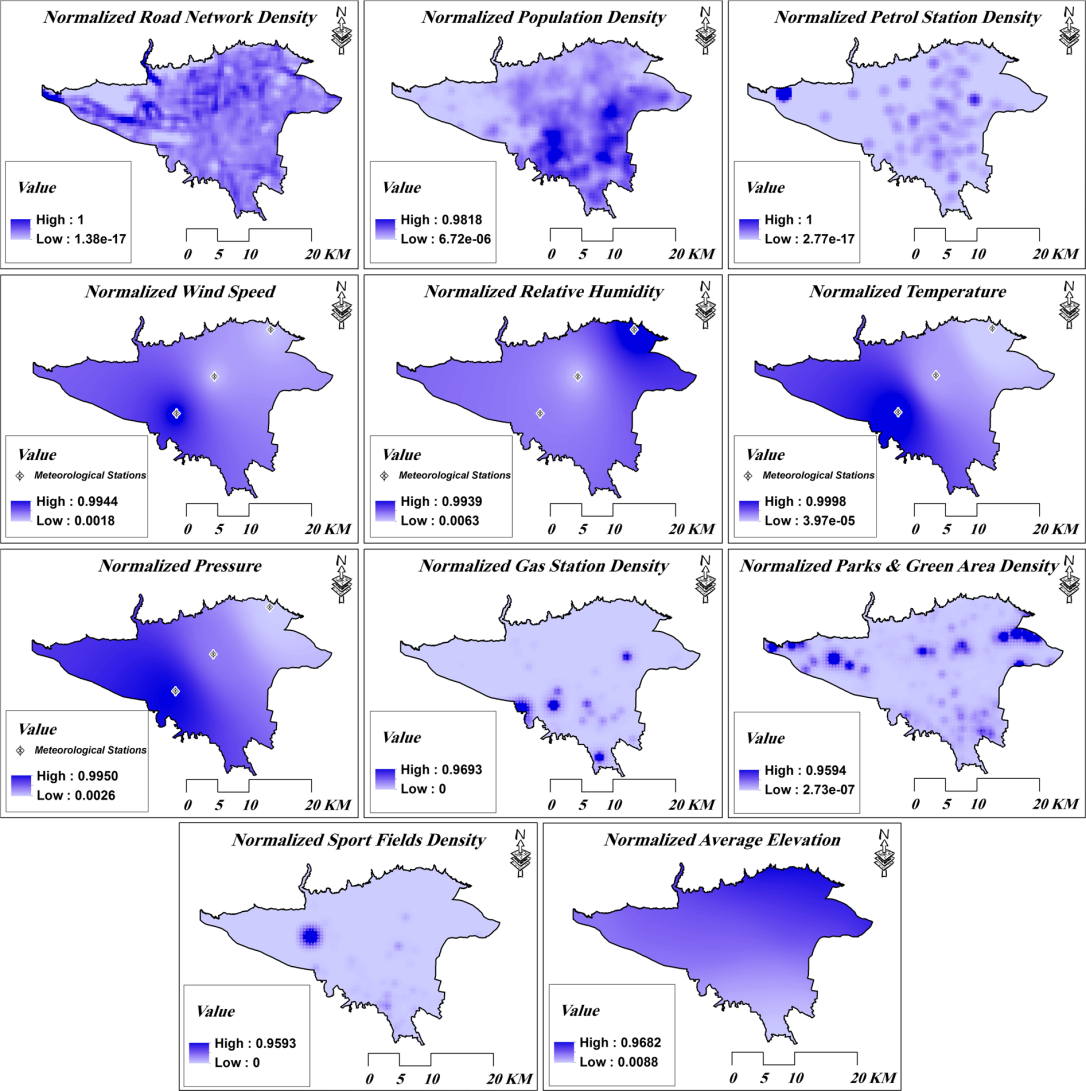
Normalized maps of input parameters on January 20, 2013

In order to validate the model, the data for 12 months, from September 2012 to August 2013, were used so that for each month; ten random times during the month were selected. For each time, the respective average hourly NO_2_ measurements of the 21 monitoring stations were retrieved from the database. The NO_2_ measurements along with the meteorological data of the respective time, elevation, POI, road network and population were feed to the model. As an example, Fig. 4 demonstrates the normalized maps of input parameters on January 20, 2013. The model was trained for that specific time and the output interpolation map was generated. Therefore, the model was trained 120 times and 120 NO_2_ interpolation maps were produced. LOOCV was used to calculate the RMSE of NO_2_ interpolation for each specific time (see “Evaluation measure”).

IDW, TPSS, OK, CK, and MARS3 along with the proposed PCAMARS model were calibrated and trained in the same condition for the selected 120 times. The input data for IDW, TPSS, and OK was latitude and longitude, but for CK and MARS3, elevation was also used as secondary data. The optimal weight for IDW and smoothing parameter for TPSS were equal to 1 and 1e+20, respectively. Furthermore, the best semi-variogram for OK and CK was spherical model. Similarly, MARS3 and PCAMARS were trained and their proper models were determined.

**Fig. 5:**
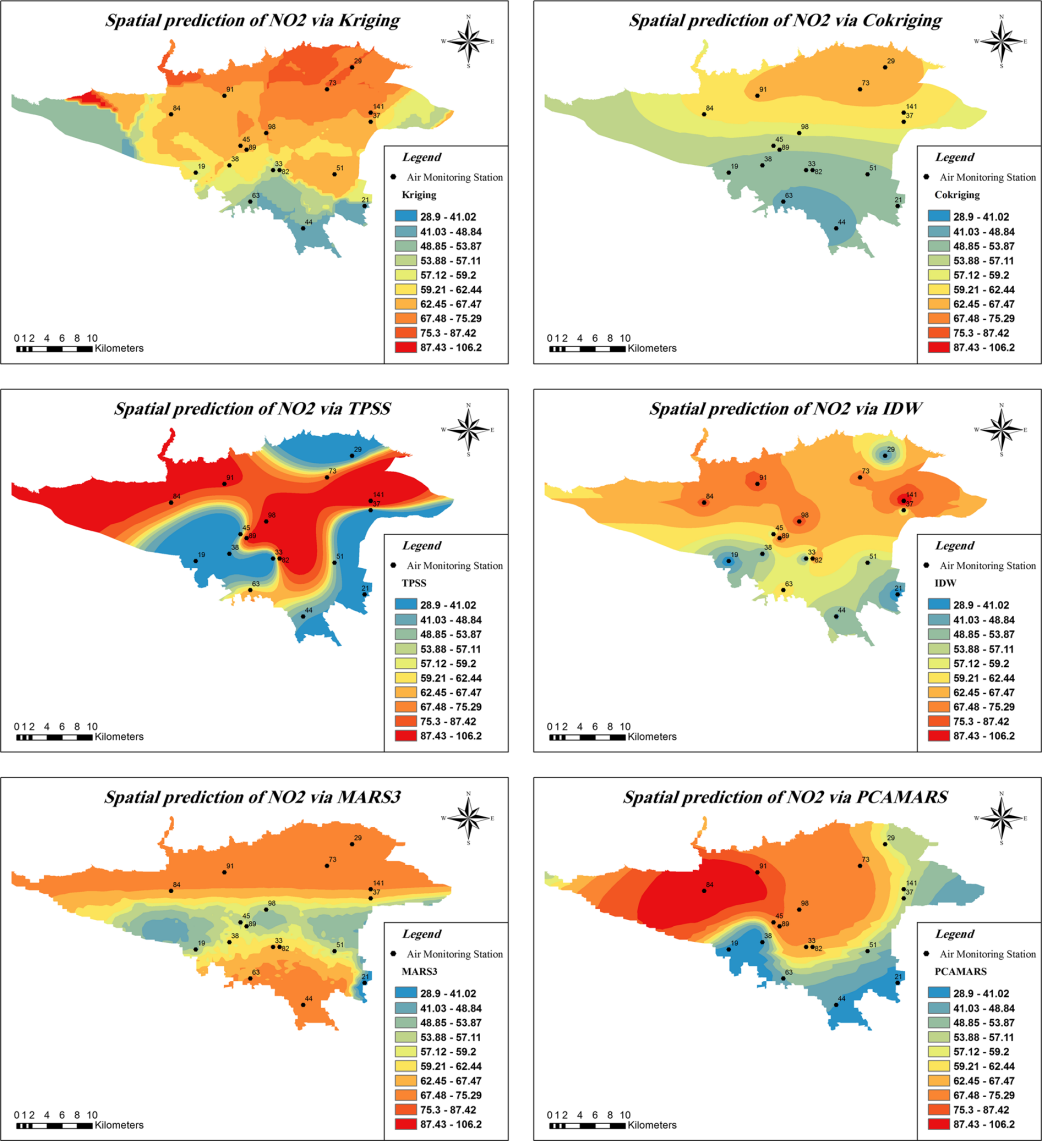
Spatial prediction of NO_2_ by all techniques on January 20, 2013

The RMSE of each model was calculated afterward, and the NO_2_ distribution map was generated for Tehran (Fig. 5). RMSE values of all techniques are shown in Tables 1 and 2. Table 1 presents the average RMSE on a monthly basis, and Table 2 shows the average RMSE of all 12 months.

**Table 1:** RMSE values

	September 2012	October 2012	November 2012	December 2012	January 2013	February 2012
IDW	28.07	27.08	41.12	30.22	26.80	38.24
TPSS	44.43	44.24	94.75	80.31	46.37	62.44
OK	25.02	25.22	33.86	25.26	24.23	34.85
CK	23.33	22.56	30.88	23.17	21.75	30.82
MARS3	21.40	20.21	28.90	18.70	20.63	30.06
PCAMARS	17.26	20.57	27.98	16.74	19.36	28.83
	March 2013	April 2013	May 2013	June 2013	July 2013	August 2013
IDW	19.67	14.05	17.45	20.15	22.76	33.70
TPSS	32.66	18.20	23.25	27.85	33.80	41.63
OK	17.24	13.54	15.67	20.93	23.44	35.98
CK	15.93	11.87	14.46	19.46	19.43	31.35
MARS3	13.47	10.15	11.50	17.78	17.20	19.56
PCAMARS	10.55	10.39	11.47	14.46	15.87	25.39

**Table 2:** Twelve-month average RMSE of methods

Method	Average RMSE
IDW	26.61
TPSS	45.83
OK	24.61
CK	22.08
MARS3	19.13
PCAMARS	18.24

According to Tables 1 and 2, the RMSE of the TPSS in comparison with other methods is peculiarly high. Based on literature, TPSS works well for the production of smooth surfaces from a large number of samples, but when large variations over short distances occur, the performance of TPSS, drops dramatically (Institute 1996). Due to the drastic changes of NO_2_ emissions in Tehran (Fig. 2), it can be concluded that TPSS is not a suitable method for interpolation of this pollutant in this city.

CK has performed better than other conventional methods. In Fig. 5, the impact of input parameters in the output of models is visible. With closer exploration of NO_2_ map, created by CK in Fig. 5, the effect of elevation can be seen. The northern part of Tehran is elevated, and as we move towards the south, this elevation gradually decreases. This change in the pattern of elevation is entirely outlined in the output of CK. On the other hand, the CK does not explicitly consider local variations through correlation parameters. For this reason, the interpolation created by CK shows a strong smoothing effect (Wang et al. 2013).

Comparing the results of the five benchmark models, namely, IDW, TPSS, OK, CK, and MARS3, showed that in most cases, MARS3 had better accuracy. This higher accuracy is a sign of the ability and capability of MARS in the domain of modeling and spatial prediction. This output also is in line with the results of Shahraiyni et al. (2015). But, as it can be seen, the supremacy of MARS3 is not absolute (Table 1). MARS3 has just three predictor variables including, latitude, longitude, and elevation which are not enough to comprehend the underlying pattern of NO_2_ distribution.

According to Tables 1 and 2, PCAMARS has shown significant superiority over the five benchmark methods. In PCAMARS, input parameters are richer in terms of data diversity. By examining the output of PCAMARS, we can see that the allocation pattern of NO_2_ has been significantly influenced by input parameters and spatial effect. This makes PCAMARS fundamentally different from other techniques. There are 3 months (October, April, and August) among 12 months in which the accuracy of MARS3 was higher than PCAMARS. It should be noted that MARS is a novel technique in the domain of interpolation, so there are not many researches about its performance and behavior. Based on the experience of the authors, one of the main reasons that could be incorporated in yielding the ascendency of MARS3 over PCAMARS, in some cases, is the number of samples in association with the number and type of predictor variables. The proposed architecture works very well especially by reducing multi-collinearity problem and can incorporate several secondary predictor variables. Another advantage of PCAMARS over MARS3 is that PCAMARS considers the spatial correlation between the dependent variable and independent variables by exploiting Moran’s I index for calculation of the parameters maps (see “Model”).

The output map of all techniques in Fig. 5 confirms each other very well. For example, in the northern and northwestern regions, there is a high level of NO_2_ emissions. There is also a decrease of NO_2_ concentration in the southern and southeastern of Tehran. It should be noted that the number of classes and the classification range of display in maps of Fig. 5 have been equalized for all techniques.

PCAMARS has the ability to consider the complex relationship between the input variables. The final output of PCAMARS for spatial modeling of NO_2_ concentration on December 21, 2013 was obtained as Eq. (6). It is notable that BF1, BF2, and BF4 only show the effects of one variable in the form of basis function for NO_2_ pollutant concentration. But BF3 represents the effect of interaction of two variables on the NO_2_ concentration, including that the intensity of BF2 is approximately two and four times more than BF1 and BF3, respectively.

6 $$ {\displaystyle \begin{array}{l}{\mathrm{NO}}_2=76.358-16.112\times \mathrm{BF}1+32.65\times \mathrm{BF}2-8.6937\times \mathrm{BF}3+44.605\times \mathrm{BF}4\\ {}\mathrm{BF}1=\max \left(0,x1+2.3324\right)\\ {}\mathrm{BF}2=\max \left(0,0.55517-x2\right)\\ {}\mathrm{BF}3=\mathrm{BF}2\times \max \left(0,2.8871-x1\right)\\ {}\mathrm{BF}4=\max \left(0,x1-2.8871\right)\end{array}} $$

Generally, the number of input parameters is not a limitation for MARS, but the results of this study indicated that incorporating a large number of predictor variables with paucity of samples, specifically when there is no precise information about the exact functional relationships among the variables, yields no satisfactory performance (Koc and Bozdogan 2015). For this reason, PCA has been employed to achieve higher accuracy.

In terms of accuracy, by utilizing PCAMARS, we are able to predict NO_2_ more accurately. The accuracy of PCAMARS combined with its low computational cost makes it a good tool to measure the exposure to NO_2_ appropriately. In this context, the authority will be able to make citizens cognizant of the density of NO_2_ in different parts of city. Therefore, citizens can reduce their exposure to NO_2_ as much as possible. This is especially relevant for vulnerable groups such as children, elderly people, asthmatics, or people suffering respiratory disease (Contreras and Ferri 2016). Additionally, the accurate maps of NO_2_ can be used as an important monitoring tool for in epidemiology studies (Robinson et al. 2013).

## Conclusion

Miscellaneous models have been proposed for interpolation and estimation of air pollution concentration. In this paper, a new model called PCAMARS has been introduced for interpolation of NO_2_ in urban areas. The proposed method is simple, accurate, and easy to implement. PCAMARS provides the ability to collectively exploit several (secondary) independent variables for interpolation of the observations of air pollution monitoring stations. Such capability is significantly important for the study areas where the number and distribution of monitoring stations are not sufficient for accurate interpolation. Additionally, the proposed model takes the spatial effect into account by considering the spatial correlation between NO_2_ and the secondary variables. The performance of the proposed model was measured against five methods, including IDW, TPSS, OK, CK, and MARS3, as standard methods, for interpolation of NO_2_ pollutant in Tehran, with an area of 730 km^2^ and only 21 monitoring stations. The results showed promising performance of PCAMARS in comparison with other methods.

As future studies, the performance of the model for interpolation of other air pollutant should be investigated thoroughly. Moreover, the accuracy of model can be improved by the utilization of more advanced dimension reduction techniques such as random forest.
